# A serum miRNAs signature for early diagnosis of bladder cancer

**DOI:** 10.1080/07853890.2023.2172206

**Published:** 2023-03-01

**Authors:** Zuhu Yu, Chong Lu, Yongqing Lai

**Affiliations:** a Department of Urology, University of Chinese Academy of Sciences-Shenzhen Hospital, Shenzhen, China; b Guangdong and Shenzhen Key Laboratory of Male Reproductive Medicine and Genetics, Peking University Shenzhen Hospital, Shenzhen, China; c The Fifth Clinical Medical College of Anhui Medical University, Hefei, China

**Keywords:** Bladder cancer, serum biomarker, diagnosis, miRNA

## Abstract

**Background:**

Bladder cancer accounts for the most common type of urologic malignancy and presents high recurrence rate after surgical resection and adjuvant intravesical therapy. We aim to search for an early diagnostic biomarker in serum for bladder cancer in this study.

**Methods:**

The expression profiles of miRNAs in serum samples of 112 bladder cancer patients and 112 healthy controls were detected with real-time polymerase chain reaction (RT-qPCR). Receiver operating characteristic (ROC) curve and area under curve (AUC) analysis were performed to assess the diagnostic efficiency of miRNAs. Stepwise logic regression analysis was used to construct a diagnostic signature with highest sensitivity and specificity. Bioinformatics analysis was applied to explore the potential biological functions and mechanisms of candidate miRNAs.

**Results:**

Five miRNAs including miR-451a, miR-381-3p, miR-223-3p, miR-142-5p and miR-27b-3p were found differentially expressed in serum samples of bladder patients and healthy subjects. The diagnostic signature was constructed with miR-27b-3p, miR-381-3p and miR-451a. AUC of the three-miRNA signature was 0.894 (0.837–0.936, *p* < 0.001). The sensitivity and specificity of this signature were 86.90% and 77.38%, respectively, indicating that this signature has a good ability to diagnose bladder cancer.

**Conclusion:**

The three-miRNA signature we constructed has favorable diagnostic capacity and may be a promising non-invasive biomarker in the early diagnosis of bladder cancer.

**KEY MESSAGES**
1. There is still no clinical utilization of serum miRNAs in the early detection of bladder cancer.
2. We screened and constructed a three-miRNA signature with the sensitivity of 86.90% and specificity of 77.38% which may be a promising non-invasive biomarker in the early diagnosis of bladder cancer.

## Introduction

Bladder cancer (BC) is the most common urologic neoplasms and generates a heavy social burden with over 200,000 related deaths worldwide annually [1]. It has many associated factors such as cigarette smoking, chronic infection or irritation, occupational exposures to polyaromatic hydrocarbons, benzene, aromatic amines and other carcinogenic chemicals [2]. Painless hematuria is the most common clinical manifestation of bladder cancer and many patients often present asymptomatic microscopic hematuria, which may lead to a delay to the diagnose [3]. Patients with non-muscle-invasive bladder cancer (NMIBC) could have their bladder saved by transurethral resection of bladder tumor (TURBT), while for patients with muscle-invasive bladder cancer (MIBC), the survival and quality of life are extremely awful after radical cystectomy and urinary diversion [4]. Early diagnosis of bladder cancer can significantly improve the survival and quality of life of patients. At present, cystoscopy remains the gold standard for bladder cancer. However, the invasiveness and corresponding postoperative complications of this inspection restrict its extensive usage in the clinical screening of cancer [5]. Thus, seeking for reliable noninvasive marker for the early diagnosis of bladder cancer is urgently needed.

MicroRNAs(miRNAs) are a group of short (about 22 nucleotides) endogenous non-coding RNAs with single molecular chain. Mature miRNAs usually regulate mRNA expression by binding to the target mRNAs, leading to mRNA degradation or translation depression [6]. Abundant evidences have proven that miRNAs play crucial role in the main features of carcinogenic process in cancers such as everlasting proliferative capacity, immune evasion, resistance to apoptosis and metastasis [7–9]. Due to the characteristics of non-invasiveness, high sensitivity, cheapness and technological maturity, serum miRNA detection is easy to large-scale clinical promotion as a new detection method. Circulating miRNAs which stably exist in peripheral blood has acquired increasing attention as their potential use of been markers in the diagnosis, prognosis and continuous monitoring of cancers [10]. Many researchers have tried to develop a non-invasive tool for the early diagnosis of cancers using miRNAs in peripheral blood [11–13]. Up to now, there is still no clinical utilization of circulating miRNAs in the early detection of bladder cancer. Hence, more circulating biomarkers with high sensitivity and specificity are needed and their relations with bladder cancer need to be probed.

In this research, we reviewed the relevant literature in PubMed with the retrieval strategies as follows: ‘Cancer’[Mesh] AND ‘MicroRNAs’[Mesh] AND ‘Screening’[Mesh] AND ‘Biomarkers’[Mesh], selected 10 most potential miRNAs for further explorations [14,15]. The selected 10 miRNAs which have been proven to be related to human cancers and have the most potential biological and diagnostic value were hsa-miR-24-3p, hsa-miR-27b-3p, hsa-miR-30c-5p, hsa-miR-128-3p, hsa-miR-142-5p, hsa-miR-206, hsa-miR-451a, hsa-miR-381-3p, hsa-miR-212-3p and hsa-miR-223-3p. We detected the expressions profile of selected miRNAs in serum samples of bladder cancer patients and healthy volunteers with RT-qPCR, obtained circulating miRNAs with diagnostic value, constructed a diagnostic signature of high sensitivity and specificity with logic regression analysis. We further predicted the target genes of the miRNAs in the signature, explored their potential functions and pathways involved in bladder cancer using bioinformatics.

## Materials and methods

### Serum specimen collection and ethic statements

The hypothesis of this study is that the diagnostic panel constructed can effectively distinguish patients from healthy controls. The research hypothesis is that the area under the ROC curve of the diagnostic panel is greater than 0.8. The area under the ROC curve of index A was 0.9 in the previous pre-experiment (or checking the literature), with *α* = 0.05 (one-sided), *β* = 0.1, and the ratio between groups was 1:1. PASS11 was used to estimate the sample size. It was found that at least 104 patients and 104 controls needed to be included. Considering a certain loss to follow-up rate, the study included 112 patients and 112 controls. The serum samples were collected from 112 patients who have diagnosed bladder urothelial carcinoma histologically and 112 healthy volunteers with no history of cancer, acute or chronic diseases from November 2017 to August 2021 in Peking University Shenzhen Hospital. The cancer group included patients with postoperative pathological findings of primary bladder cancer who had not received immunotherapy and other therapies. Age and gender of healthy controls were matched to patients with bladder cancer. All the participants signed the informed consent before sample acquisition. This study was approved by the Ethics Committee of Peking University Shenzhen Hospital and serum sample collection followed relevant rules of the committee. The serum samples were centrifuged at 1000 g for 10 min and 15,000 g for 5 min at 4 °C within 2 h after isolated from participants. Serum was then stored at −80 °C pending further processing.

### Experiment design

Firstly, we conducted the screening phase. We used RT-qPCR to detect the expression levels of 10 selected miRNAs in 28 serum samples from BC patients and 28 healthy control (HC) serum samples and selected the required miRNAs for the next phase. The standard of candidate miRNA was *p* < 0.05. Subsequently, we verified the expression level of candidate miRNAs in the validation phase using another 84 BC patients and 84 HCs. Lastly, we performed backward stepwise logistic regression analysis to construct a diagnostic miRNAs signature and receiver operating characteristic (ROC) curve to calculate the area under the curve (AUC).

### RNA extraction and RT-qPCR

To normalize the extraction and purification process and control the variability of study, 2 µL of synthetic Caenorhabditis Elegans miRNA-39 (RiboBio, Guangzhou, China) was added into each serum sample as an internal control RNA [16]. Total RNA of each serum sample was extracted using a TRIzol LS isolation kit (Thermo Fisher Scientific, Waltham, USA), resuspended with 30 µL RNase-free water and stored at −80 °C conforming to the manufacturer’s instructions. Concentration and purity of RNA in were measured using a NanoDrop 2000 spectrophotometer (Thermo Scientific, USA).

The amplification of miRNAs was proceeded using the reverse transcription specific primers of the Bulge-LoopmiRNA qRT-PCR Primer Set (RiboBio, Guangzhou, China). The real-time polymerase chain reaction (RT-qPCR) was performed on the LightCycler480 Real-Time PCR system (Roche Diagnostics, Mannheim, Germany), using an SYBR Green qPCR Kit (SYBR Pre-mix Ex Taq II, TaKaRa, Japan). Parameters of the reaction was set as 95 °C for 20 s, following 40 cycles of 95 °C for 10 s, 60 °C for 20 s and 70 °C for 10 s.

### Statistical analysis

The relative expressions of miRNAs were normalized to the internal control cel-miR-39 and calculated with 2^−ΔΔCt^ method. Student’s *t* test or χ^2^ test was performed to test the differences of miRNAs expression levels in BC and HCs samples. Multiple logistic regression analysis was used to build the miRNA signature and Hosmer Lemeshow goodness-of-fit test was used to evaluate the calibration. Receiver operating characteristic (ROC) curves, the area under the ROC curve (AUC) and Youden index were used to assess the diagnostic value of miRNAs and determine the most effective diagnostic signature with the highest sensitivity and specificity. Statistical analyses software like SPSS (Version 20), GraphPad Prism (Version 8) and MedCalc (Version 19) were used in this study. *p* < 0.05 was defined as statistically significant.

### Bioinformatic analysis

We performed target prediction of candidate miRNAs using miRDB (http://mirdb.org/) miRTarBase (https://maayanlab.cloud/Harmonizome/resource/MiRTarBase) and TargetScan (http://www.targetscan.org/vert_80/) synchronously, took the intersection as the candidate targets of miRNAs. The STRING database (https://www.string-db.org/, version 11.0) was utilized to explore the interactions of target genes with the minimum required interaction score of 0.4. Cytoscape (version 3.9.1) was applied for visualizing molecular interaction network of target genes and the plug-in cytoHubba was ran for identifying the hub genes and subnetworks. The hub genes with top 10 MMC value were selected and validated in GEPIA (http://gepia.cancer-pku.cn/). Gene ontology analysis (GO) and Kyoto Encyclopedia of Genome (KEGG) annotation of target genes were conducted with Enrichr database (http://amp.pharm.mssm.edu/Enrichr/).

## Results

### Participants’ characteristics involved in this research

In total, 112 bladder cancer patients and 112 healthy controls (HCs) participated in this research. The histological classification of the patients was based on the standards of the World Health Organization and all patients were confirmed by the TNM staging system. All healthy controls had no history of cancer or other diseases. Statistic and clinical characteristics of the participants are listed in Table 1. No significant differences in gender and age were observed between BC patients and healthy controls.

**Table 1.** Clinical characteristics of the 224 participants in this research.

	BC patients	HCs
Total number	112	112
Age (mean ± SD)	61.8 ± 13.6	59.3 ± 13.1
Gender (%)		
Male	91 (81.2)	85 (75.9)
Female	21 (18.8)	27 (24.1)
T stage (%)		
Ta	24 (21.4)	
T1	60 (53.6)	
T2	20 (17.9)	
T3–4	8 (7.1)	
Grade (%)		
Low	58 (51.8)	
High	54 (49.2)	

### Screening of candidate miRNAs in the screening phase

**Figure 1.**
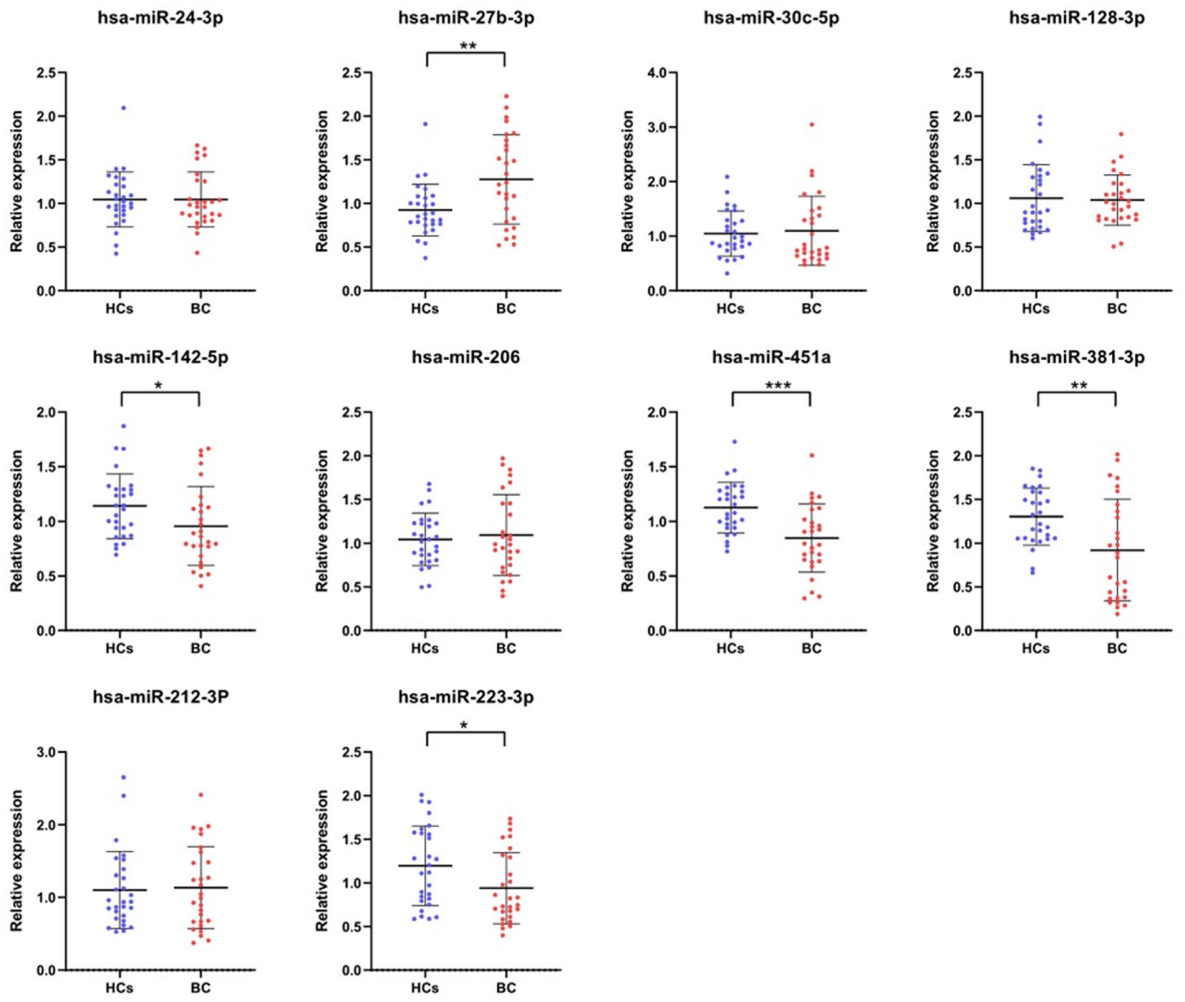
The expression levels of 10 candidate miRNAs in the training phase. Screening conditions: *p*-value <0.05, * represents *p* < 0.05, ** represents *p* < 0.01, *** represents *p* < 0.001.

In the screening phase, we detected the expression of 10 candidate miRNAs from literature in 28 serum samples of bladder cancer patients and 28 healthy controls with RT-qPCR. The results are shown in Figure 1. With the filter criteria of *p* < 0.05, five candidate miRNAs (miR-451a, miR-381-3p, miR-223-3p, miR-142-5p, miR-27b-3p) were picked out for further validation and analysis.

### Verification of candidate miRNAs in the validation phase

**Figure 2.**
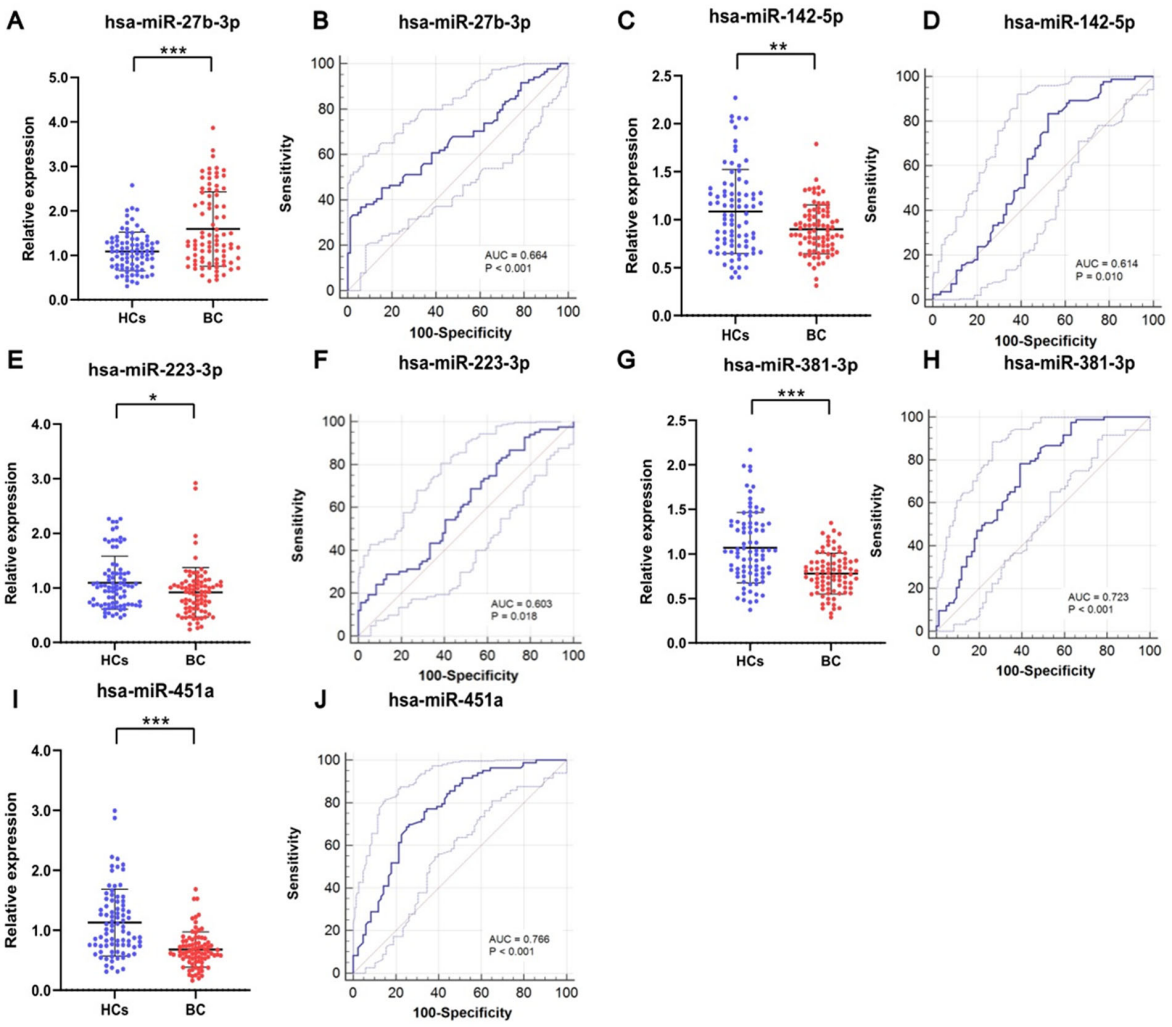
The relative expression of 5 candidate miRNAs and the corresponding ROC curves. The expression of miR-142-5p (C), miR-223-3p (E), miR-381-3p (G) and miR-451a (I) in serum sample of bladder cancer were significantly lower than in HCs. The expression of miR-27b-3p in cancerous serum samples was significantly higher than in HCs. AUCs of miR-27b-3p (B), miR-142-5p (D), miR-223-3p (F), miR-381-3p (H) and miR-451a (J) were 0.664 (*p* < 0.001), 0.614 (*p* = 0.010), 0.603 (*p* = 0.018), 0.723 (*p* < 0.001) and 0.766 (*p* < 0.001), respectively. * *p* < 0.05, ** *p* < 0.01, *** *p* < 0.001.

To determine the expression of 5 candidate miRNAs in serum sample of BC patients and HCs, we further detected their expression levels in serum samples of 84 BC patients and 84 HCs by amplifying sample capacity. As shown in Figure 2, the relative expressions of the 5 miRNAs in the serum of BC patients and HCs were significant different (*p* < 0.05). The expression levels of miR-142-5p, miR-223-3p, miR-381-3p and miR-451a in serum sample of patients with bladder cancer were significantly lower than in HCs, while miR-27b-3p showed significant higher expression in serum samples of BC patients than in healthy volunteers (Table 2).

**Table 2.** The mean fold change of miRNAs expression in all BC patients and healthy controls.

miRNAs	BC patients	Healthy controls	*p* Value
hsa-miR-27b-3p	1.593 ± 0.836	1.078 ± 0.434	<0.001
hsa-miR-142-5p	0.899 ± 0.254	1.085 ± 0.483	<0.01
hsa-miR-223-3p	0.918 ± 0.453	1.095 ± 0.487	<0.05
hsa-miR-381-3p	0.780 ± 0.229	1.072 ± 0.394	<0.001
hsa-miR-451a	0.676 ± 0.294	1.126 ± 0.557	<0.001

### Diagnostic value assessment of the candidate miRNAs

To assess the diagnostic value of being noninvasive markers for bladder cancer, we draw ROC curves of candidate miRNAs, calculated the AUC and Youden index. The results of the candidates were shown in Figure 2 and Table 3. The AUCs of miR-27b-3p, miR-142-5p, miR-223-3p, miR-381-3p and miR-451a were 0.664 (0.588-0.735, *p* < 0.001), 0.614 (0.536–0.688, *p* = 0.010), 0.603 (0.524–0.677, *p* = 0.018), 0.723 (0.650–0.790, *p* < 0.001) and 0.766 (0.693–0.826, *p* < 0.001), respectively. As shown in Table 3, Youden index indicated that miR-142-5p, miR-223-3p and miR-381-3p have high sensitivity (83.33%, 80.95%, 78.57% respectively) while miR-451a and miR-27b-3p have high specificity (75.00% and 94.05%).

**Table 3.** Results of receiver operating characteristic curves and Youden index for 5 candidate miRNAs and the three-miRNA panel.

	AUC	*p* Value	95% CI	Associated criterion	Sensitivity (%)	Specificity (%)
miR-142-5p	0.614	0.010	0.536–0.688	≤1.12	83.33	47.62
miR-223-3p	0.603	0.018	0.524–0.677	≤1.13	80.95	35.71
miR-381-3p	0.723	<0.001	0.650–0.790	≤0.92	78.57	60.71
miR-451a	0.766	<0.001	0.693–0.826	≤0.74	67.86	75.00
miR-27b-3p	0.664	<0.001	0.588–0.735	>1.81	36.90	94.05
three-miRNA panel	0.894	<0.001	0.837–0.936	>0.38	86.90	77.38

AUC: area under curve; CI: confidence interval.

### Construction of the diagnostic miRNAs signature for bladder cancer

**Figure 3.**
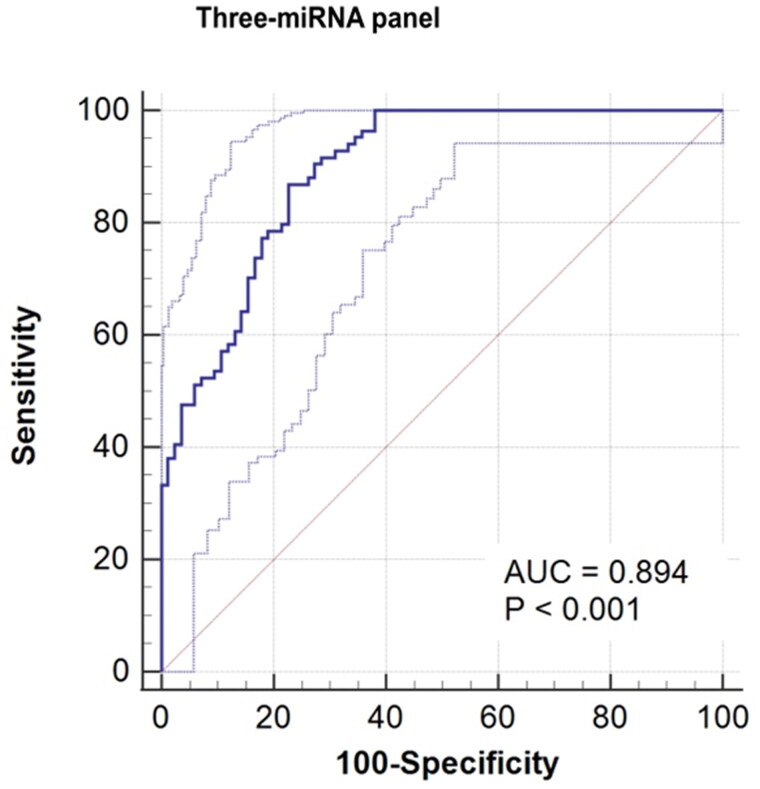
Receiver operating characteristic (ROC) curve of the three-miRNA signature including miR-27b-3p, miR-381-3p and miR-451a. The AUC of the signature is 0.894 (95%CI: 0.837-0.936, *p* < 0.001).

In order to determine the most effective diagnostic signature with highest sensitivity and specificity, we constructed a three-miRNA panel with miR-27b-3p, miR-381-3p and miR-451a using stepwise logic regression analysis. The calculation formula of the model is Logit(P)= 2.831+(2.182 × expmiR-27b-3p)+(−3.311 × expmiR-381-3p)+(−2.983 × expmiR-451a). As shown in Figure 3, AUC of the three-miRNA panel was boost to 0.894 (0.837–0.936, *p* < 0.001). Accordingly, the sensitivity and specificity of the model was enhanced to 86.90% and 77.38% respectively (Table 3), suggesting the model has attractive diagnostic capacity of distinguishing bladder cancer and healthy controls.

### Target prediction of candidate miRNAs and bioinformatic analysis

**Figure 4.**
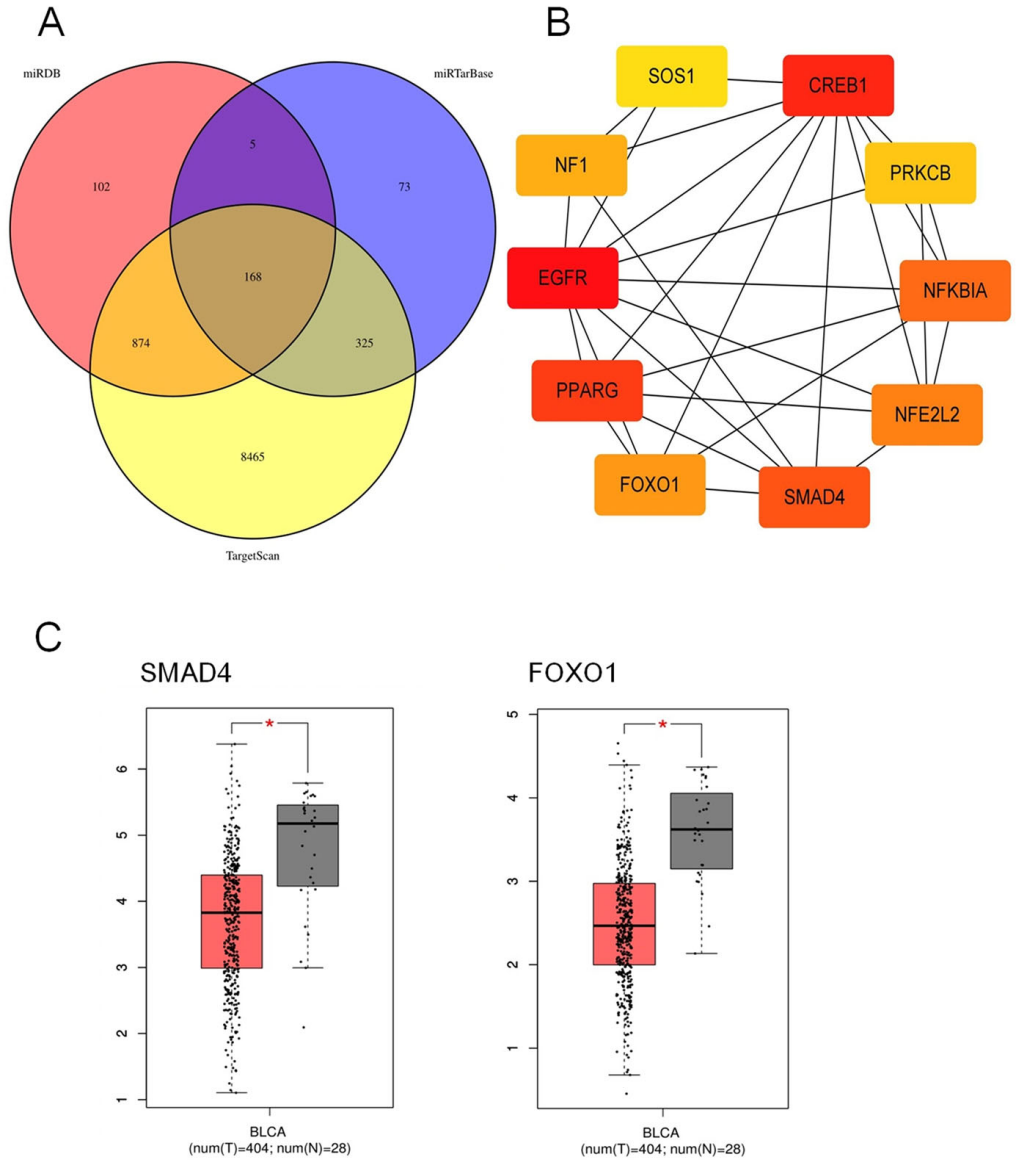
Target prediction of the miRNAs in the signature. (A) In total, 168 target genes of the three miRNAs were predicted by miRDB, miRTarBase and TargetScan simultaneously. (B) The subnetwork and top ten hub genes obtained using CytoHubba of Cytoscape. (C) GEPIA database showed that SMAD4 and FOXO1 were down-regulated in bladder cancer tissues compared to normal tissues (*p* < 0.05). BLCA: Bladder Urothelial carcinoma. T: Tumor tissues. N: Normal tissues. * *p* < 0.05.

In order to analyses the function of miRNAs in the diagnostic signature and probe their possible involved signaling pathways in the carcinogenesis and progression of bladder cancer, we executed target prediction of the 3 miRNAs in the signature with miRDB, miRTarBase and TargetScan. In total, we harvested 168 target genes of these miRNAs by taking the intersection, as displayed in Figure 4(A). We implemented protein-protein interaction analysis with STRING database and visualized the network with Cytoscape. The subnetwork and top ten hub genes obtained by CytoHubba plug-in are shown in Figure 4(B). We further explored the expression of the hub genes in bladder cancer tissues and normal tissues with GEPIA. The results indicated that SMAD4 and FOXO1 were down-regulated in bladder cancer tissues compared to normal tissues (*p* < 0.05) (Figure 4(C)).

**Figure 5.**
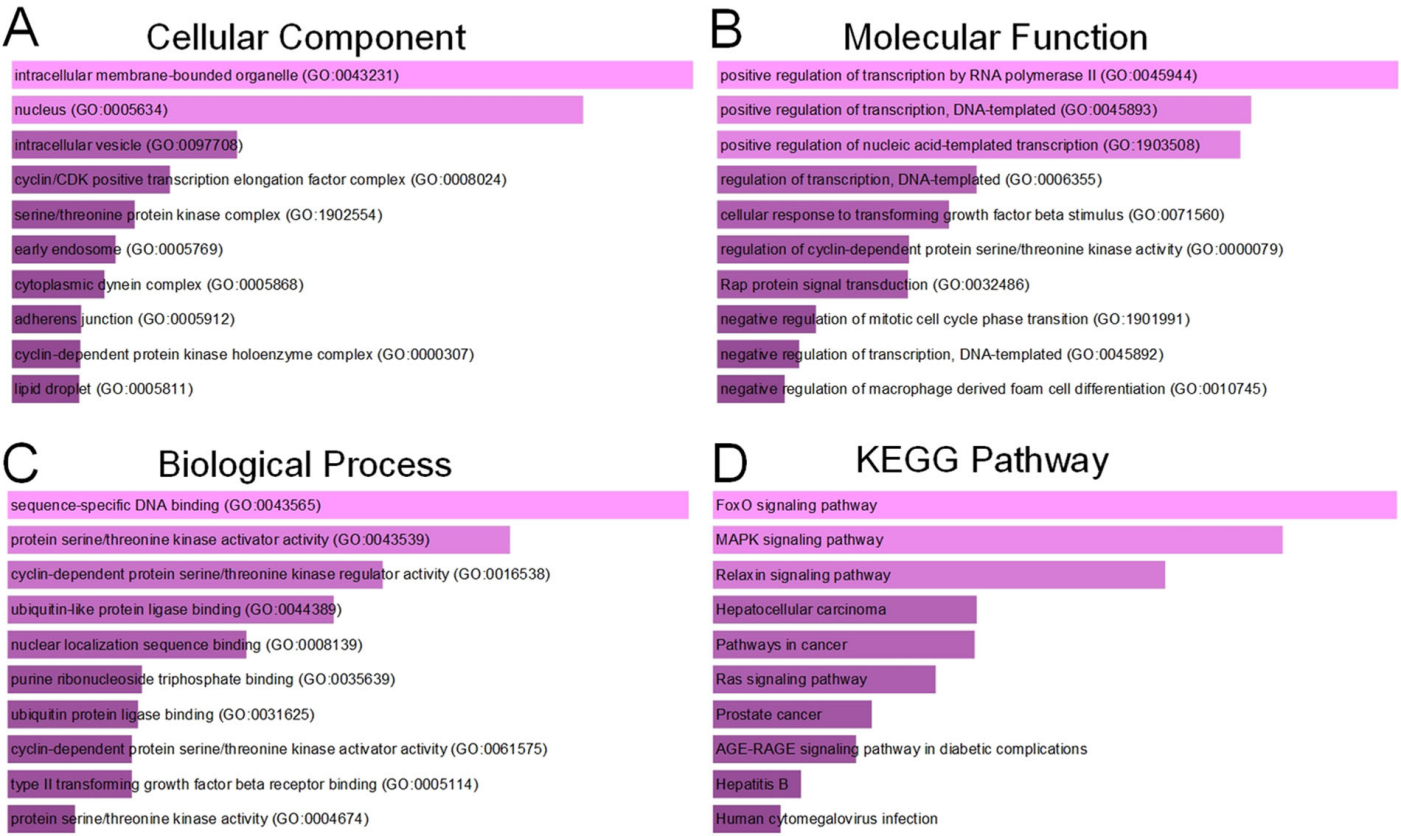
GO annotations and KEGG pathway enrichment analysis of the target genes of miR-27b-3p, miR-381-3p and miR-451a. (A) Cellular component enrichment; (B) Molecular function enrichment; (C) Biological Process enrichment; (D) KEGG Pathway enrichment analysis.

The function annotation of the target genes was analyzed with Enrichr database and illustrated in Figure 5. Go analysis revealed that these genes were mostly enriched in intracellular membrane-bounded organelle, nucleus, intracellular vesicle, cyclin/CDK positive transcription elongation factor complex, serine/threonine kinase complex. Molecular function of the target genes was enriched in regulation of transcription, cellular response to transforming growth factor beta stimulus. Biological process of the target genes involved was enriched in sequence-specific DNA binding, protein serine/threonine kinase activator activity, cyclin-dependent protein serine/threonine kinase activator activity, ubiquitin-like protein ligase binding. KEGG pathway analysis indicated the target genes enriched in FOXO signaling pathway, MAPK signaling pathway, Relaxin signaling pathway, Hepatocellular carcinoma, pathways in cancer. The functional annotation implied that target genes of the three miRNAs may be involved in proliferation and progression of bladder cancer.

## Discussion

For decades, the diagnosis, treatment and prognosis of bladder cancer have not been improved satisfactorily. Like other malignancies, early diagnosis can significantly improve the survival and quality of life of BC patients by avoiding losing the chance of surgery or disturbing complications after radical cystectomy. The existing diagnostic methods are characterized by low accuracy or invasiveness, which cannot meet the requirements of early screening of BC well [17]. Circulating miRNAs have been confirmed stably existed in blood and are related to certain diseases including tumors, making it possible to use peripheral blood to diagnose or monitor cancers [18]. In the study of BC, some biomarkers have been found potentially useful in early diagnosis [19]. However, no useful miRNA markers are recommended as diagnostic tool in the clinical detections of the inconsistencies of the results among these studies [20]. Except for the preanalytical and analytical variables as well as donor-related factors, the function and mechanism of these miRNAs in BC have not been verified, which is also an important reason.

In the present study, we screened 10 cancer-related miRNAs which have been verified in previous studies, used RT-qPCR to detect their expression levels in serum of BC patients and healthy participants, obtained 5 miRNAs (miR-451a, miR-381-3p, miR-223-3p, miR-142-5p, miR-27b-3p) which differentially expressed. We further validated their expression levels with an enlarged sample pool and assessed the capacity of these miRNAs as diagnostic biomarkers of BC. Using regression analysis, we constructed a miRNAs panel of high sensitivity and specificity with miR-27b-3p, miR-381-3p and miR-451a. This miRNAs signature has favorable ability of identifying bladder cancer patient and healthy subject. Up to now, no studies have investigated the relationship between the three miRNAs (miR-27b-3p, miR-381-3p, miR-451a) and bladder cancer. Therefore, we are the first to discover that the three miRNAs were differentially expressed in peripheral blood of bladder patients and healthy subjects and to construct a panel using the three miRNAs for the early diagnosis of bladder cancer.

Numerous researches have demonstrated that miR-27b-3p engages in the regulation pathways of many cancers. For example, miR-27b-3p were found to act as tumor suppressor in glioma, gastric cancer, lung cancer, endometrial carcinoma and esophageal cancer [21–23]. But the studies in colorectal cancer and breast cancer showed contradictory results [24–27]. The expression and function of miR-27b-3p in bladder cancer have not been studied formerly. Hence, we firstly revealed that the expression levels of miR-27b-3p were significantly over-expressed in serum of BC patients compared to healthy subjects in this study. Existing studies on the function of miR-381-3p in tumors have shown spectacular consistency as they all indicated that this miRNA plays the role of tumor suppressor in various cancers, including BC [28,29]. A research of bladder cancer *in vitro* showed miR-381-3p inhibits the progression of bladder cancer cells by binding to RAB2A (Ras-Related Protein Rab-2A). Another experiment also proved that miR-381-3p blocks the dual regulatory role of CCNA2 (Cyclin-A2) in modulating CDK6 and MET-mediated cell-cycle pathway and EMT progression in bladder cancer [30,31]. Identical to miR-381-3p, miR-451a acts as anti-cancer factor in various cancer and inhibits the proliferation, migration and invasion of cancer cells [32,33]. In this research, the expressions of miR-381-3p and miR-451a in serum of BC patients were both lower than in healthy controls, which is consistent with previous studies.

Furthermore, we predicted the target genes of the miRNAs panel, explored the function and mechanism of these miRNA involved in the tumorigenesis and progression of BC, and discovered some meaningful results. The bioinformatic analysis suggests that SMAD4 and FOXO1 are the target genes of the miRNAs in the signature, they are down-regulated in bladder tissues compared to normal tissues. The results are confirmed by previous researches. Abundant studies have proven that SMAD4 loss can promote tumor progression in different types of tumors, including bladder cancer [34,35]. FOXO1 has been substantiated as a tumor suppressor gene in cancers and its expression level is positively correlated with the prognosis of BC patients [36,37]. Therefore, we surmise that miR-27b-3p, miR-381-3p and miR-451a play important roles in the occurrence and progression of BC by targeting SMAD4 and FOXO1. The Go and KEGG analysis of the target genes also supports this assessment.

The present study also has certain limitations. First, the experimental results of this study are retrospective, so prospective and multicenter studies are needed to verify the accuracy and availability of diagnostic features. In addition, our study only considered the expression level of miRNA in serum, without further verifying whether their expression is consistent in histology, which requires further experiments to improve our conclusion. Finally, we did not consider whether the patients had other prognostic and risk factors when we collected them, which should be supplemented and improved in our later experiments.

## Conclusion

In this study, we constructed a diagnostic signature of bladder cancer with miR-27b-3p, miR-381-3p and miR-451a, which were found differentially expressed in serum samples of BC patients and healthy subjects. The three-miRNA signature has favorable diagnostic ability to identify bladder cancer and may be a promising non-invasive biomarker in the early diagnosis of bladder cancer.

## Data Availability

The data used in the present study are available from the corresponding authors upon reasonable request.
